# Life cycle assessment of biocemented sands using enzyme induced carbonate precipitation (EICP) for soil stabilization applications

**DOI:** 10.1038/s41598-022-09723-7

**Published:** 2022-04-11

**Authors:** Emran Alotaibi, Mohamed G. Arab, Mohamed Abdallah, Nadia Nassif, Maher Omar

**Affiliations:** 1 Department of Civil and Environmental Engineering, University of Sharjah, P.O. Box 27272, Sharjah, United Arab Emirates; 2 Research Institute of Sciences and Engineering, University of Sharjah, Sharjah, United Arab Emirates; 3 Structural Engineering Department, Mansoura University, Mansoura, Egypt

**Keywords:** Biogeochemistry, Environmental sciences, Environmental impact

## Abstract

Integrating sustainability goals into the selection of suitable soil stabilization techniques is a global trend. Several bio-inspired and bio-mediated soil stabilization techniques have been recently investigated as sustainable alternatives for traditional techniques known for their high carbon footprint. Enzyme Induced Carbonate Precipitation (EICP) is an emerging bio-inspired soil stabilization technology that is based on the hydrolysis of urea to precipitate carbonates that cement sand particles. A life cycle assessment (LCA) study was conducted to compare the use of traditional soil stabilization using Portland cement (PC) with bio-cementation via EICP over a range of environmental impacts. The LCA results revealed that EICP soil treatment has nearly 90% less abiotic depletion potential and 3% less global warming potential compared to PC in soil stabilization. In contrast, EICP in soil stabilization has higher acidification and eutrophication potentials compared to PC due to byproducts during the hydrolysis process. The sensitivity analysis of EICP emissions showed that reducing and controlling the EICP process emissions and using waste non-fate milk has resulted in significantly fewer impacts compared to the EICP baseline scenario. Moreover, a comparative analysis was conducted between EICP, PC, and Microbial Induced Carbonate Precipitation (MICP) to study the effect of treated soil compressive strength on the LCA findings. The analysis suggested that EICP is potentially a better environmental option, in terms of its carbon footprint, at lower compressive strength of the treated soils.

## Introduction

Historically infrastructure projects, that involve soil stabilization due to challenges related to the soil conditions, are evaluated based on performance and cost. Recently, environmental and social sustainability has been introduced as important elements in the decision-making process in these soil stabilization projects^1,2^. Using Portland cement (PC) as a soil stabilizer has been a common practice in geotechnical engineering for several decades^3^. PC applications for soil stabilization have shown economical and performance benefits despite durability concerns especially in sulfate contaminated soils^4^. The mechanical properties of PC-treated soils have been well studied in the literature^5,6^.

Despite economical and performance advantages of PC, its production is responsible for approximately 5–7% of total carbon dioxide (CO_2_) emissions worldwide^7^. Emissions from PC plants also include sulfur dioxide (SO_2_) and nitrous oxides (NO_x_) which contribute to acid rain and global warming. In addition, PC production contributes to the consumption of significant quantities of natural resources; 1.5 tons of raw materials are required to produce one ton of PC^8^. Furthermore, clinker manufacturing involves massive energy consumption^9^.

Recent developments in the use of biologically based approaches for soil stabilization are believed to be sustainable alternatives to PC. Among these techniques is the bio-cementation using carbonate precipitation utilizing hydrolysis of urea. In the hydrolysis process reaction, urea solution (CO(NH_2_)_2_) is hydrolyzed into carbonate (CO_3_^2−^) and ammonia (NH_4_^+^) ions in the presence of calcium ion (Ca^2+^) source usually calcium chloride (CaCl_2_); resulting in ammonium chloride (NH_4_Cl) and calcium carbonate (CaCO_3_). This reaction is catalyzed by urease enzymes derived from plant or bacterial sources. In 2004, Whiffin^10^ proposed the use of sporosarcina pasteurii bacteria as a source of urease enzyme for carbonate soil treatment. This technique is referred to as microbial-induced carbonate precipitation (MICP). Several potential MICP applications have been proposed for improving granular soils^11–19^. However, one of the key MICP limitations is the mode of application that requires multiple two-phase cycles of treatment to achieve sufficient strength and carbonate precipitation^20,21^. In addition, the MICP process uses bacteria that often require a suitable and sensitive environment for growth and enzyme production^22–24^.

Recently, free urease enzyme derived from plant sources was suggested as a catalyst in hydrolysis. This hydrolysis technique is usually referred to as enzyme-induced carbonate precipitation (EICP). EICP is a bio-inspired ground improvement technique that has been recently investigated in multiple applications including soil stabilization^10,13,25–28^, dust control^29–35^, and water erosion resistance^36^. Several researchers have shown the successful application of EICP through mixing the EICP cementing solution with sand and compaction, similar to conventional subgrade soil treatment for pavement applications^25,26,28,37^. However, this mode of application (mix and compact) was reported for a few studies that utilized MICP to improve shear strengths of clay soils of intermediate and high plasticity^38,39^. MICP is mostly used as cementing binder for permeation grouting and soil injection for sandy soils^11,40,41^.

Recently, Almajed et al.^28^ and Martin et al.^42^ have reported improved performance of EICP treated soils via mix and compact by adding non-fat milk powder to the solution. These biologically driven techniques are still in the early development stages and are not yet examined for large field or commercial usage. Therefore, it is important to critically evaluate all the outputs from these processes in order to enhance its economy and sustainability. For example, in EICP treatment chloride ions may combine with the ammonium ions to make ammonium chloride, a salt the environmental impact of which may be of concern. Also, part of the ammonium ions may volatilize as NH_3_^43^. To date, detailed experimental measurements of EICP process emissions have not been carried out in the literature.

Life cycle assessment (LCA) is a systematic tool utilized to evaluate potential environmental impacts and resources used throughout a product’s lifecycle, i.e., from material extraction, through production and application phases, to ultimate disposal^44^. LCA has been conducted extensively in the literature on cement production and its environmental impacts with soil mixing^45^. On the other hand, the LCA of bio-cementation techniques for soil stabilization, in general, has been scarce in the literature. Deng et al.^46^ used a quantitative relationship between the compressive strength of MICP-treated sand and the carbonate content as a basis for the LCA analysis of MICP for soil stabilization based on the compressive strength level. The authors investigated the energy consumption and carbon emission of MICP-treated sand compared to PC concrete, sintered bricks, PC treated backfill, and cement grouting. For each application, a target strength was set based on typical values. It was concluded that MICP offers better environmental performance in cases that require low compressive strength such as treated backfill, since greater strengths of MICP-treated sand require higher carbonate precipitation, and hence, a higher number of treatment cycles. EICP soil stabilization technique is based on hydrolysis, similar to MICP, without the need for bacterial activity. Raymond et al.^47^ conducted a life cycle sustainability assessment on EICP as a dust suppressant compared to common dust mitigation strategies. They concluded that EICP is more sustainable than watering, where the main factor was the frequency of water applications needed compared with the durability of EICP one application. In addition, their results showed that EICP is more costly and environmentally intensive compared to MgCl_2_ used as a dust suppressant and that it can be potentially more sustainable by including the long-term performance of EICP treatment. Martin et al.^48^ performed an LCA on EICP for sand columns improvement. They found that the largest contributors for equivalent carbon emissions were nonfat milk powder and urea with 38 and 35%, respectively. The study did not include a comparison with conventional techniques for soil stabilization such as PC.

In light of the above knowledge gaps in the literature, this paper presents a comparative LCA for EICP and PC for soil stabilization to quantify their life cycle environmental impacts. The specific objectives were to: (1) identify the process and streams of PC and EICP processes, (2) compute the environmental impacts based on the various emissions generated, and (3) conduct sensitivity analysis of the examined scenarios to assess the effects of input variation and uncertainty propagation. This research is one of the first studies that conduct a comparative study between the soil stabilization utilizing EICP and conventional soil stabilization using PC. Moreover, a sensitivity analysis was conducted to study the effect of target compressive strength of EICP treated sand with sand treated with PC and MICP. The research findings are essential to direct the ongoing research effort towards improving the environmental performance of EICP throughout its life cycle.

## Methodology

The LCA methodology conducted in this study was based on the International Organization for Standards (ISO) guidelines 14,040–14,044^44^. The environmental impacts of the EICP and PC production and application processes were evaluated through four stages: (1) definition of scope and system boundaries, (2) quantification of processes and their inputs/outputs inventories, (3) assessment of inventory data; and (4) results from interpretation and improvement recommendations.

### Goal and scope definition

The goal of this study was to conduct an LCA to investigate and compare the environmental impacts of EICP against PC for soil stabilization. The functional unit (FU) selected for comparing both systems was a poorly graded native soil area of 10,000 m^2^ (25 m by 400 m) to serve as an unpaved road for light vehicles. The target performance parameter was used in this study an average UCS of 1.5 MPa for treated subgrade soil in a 2-week period for a 150-mm thick layer. This target was established based on typical values for target values for unpaved road applications^49^. The project site is located in Dubai, United Arab Emirates (Northing: 25° 06′ 56″, Easting: 55° 10′ 08″). Dubai has an average winter and summer temperatures of 19.5 and 35.4 °C, respectively. The study area was characterized by homogenous sand with flat formation.

### Life cycle inventory

#### Data inventory and materials

The mixing ratios suggested by Almajed et al.^26^ for urea, calcium chloride, milk, and urease were used to achieve the target UCS of 1.5 MPa. The selected EICP solution consisted of 30, 1.82, 2.23, 0.121, and 0.091% of water, urea, CaCl_2_, non-fat milk powder, and Jack bean meal (urease), respectively, by weight of dry soil. Enzyme, urea, CaCl_2,_ and non-fat milk were assumed to be available from local suppliers (40 km from the study area).

In Almajed et al.^26^ study, similar soil under the same relative density was treated with 14.0% cement by weight of dry sand to reach the same target UCS of 1.5 MPa with a water to cement ratio of 1:1.5. The cement was assumed to be supplied from a cement production facility located 20 km away from the study area. Water was assumed to be supplied through a tanker delivering the water from a potable water source located 20 km away from the project site. During the construction of cement treated subgrade a saturated wet covering technique was assumed to avoid cement shrinkage during the initial setting period^50^. The application of this method was assumed by spraying water over the surface of the study area (0.01 m^3 `^water/m^2^ of surface area)^51^.

**Figure 1:**
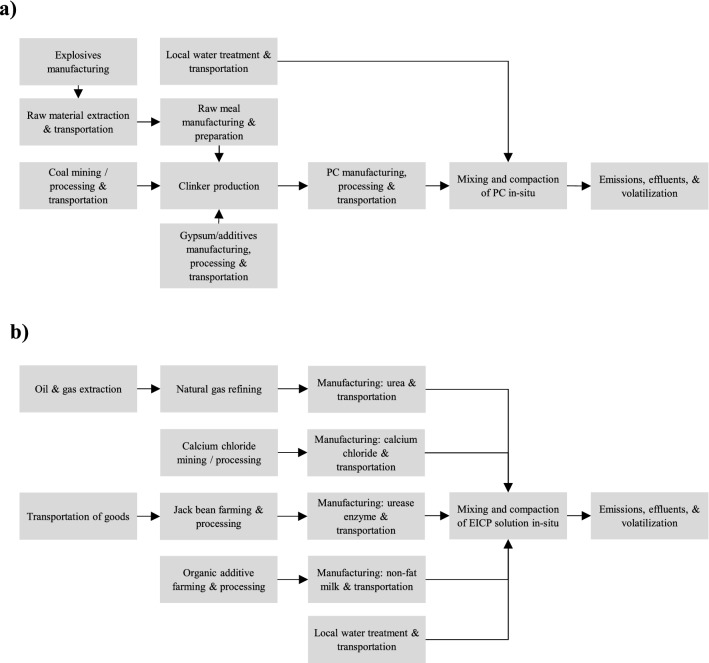
Definition of system boundary for the (**a**) PC and (**b**) EICP soil stabilization techniques.

The inventory data for the production processes of PC, milk, urea, and CaCl_2_, as well as the transportation and application processes, were extracted from the Ecoinvent 3.0 international database and adjusted according to the required scope and conditions. A detailed description of the urea production process and material flows used in this study are summarized in Appendix 1. As the latest version of the Ecoinvent database did not include farming and processing of jack beans, this process was replaced by soybean data which is considered nearly similar in agricultural and manufacturing processes^48^. During the process, the Jack beans were assumed to be deshelled then purified by adding acetone and acid then centrifuged^52^. After one purification cycle of deshelled jack beans, similar specific enzymatic activity for the application needed in the present study was obtained^52^. A 14% loss in the weight of jack beans due to deshelling is adopted in this study. The system boundary of the PC and EICP processes are shown in Fig. 1; almost all processes were included except for the manufacturing of equipment in factories. Inputs for the transportation processes of water and materials constituted the distances traveled by the freight lorries (ton-kilometer) of 32-metric ton capacity and their fuel consumption.

#### Emissions from EICP and PC

The air and soil emissions from the EICP and PC application were included in this study. The onsite urea emissions were estimated as per the recommendations of the Intergovernmental Panel on Climate Change (IPCC) based on previous studies that quantified the emissions of fertilizers^53^. IPCC recommendations were deemed the best available method for estimating urea on-site emissions in the absence of field data^43^. The IPCC suggests an emission factor of 1% of the applied Nitrogen (N) to estimate the direct N_2_O released from fertilized soils. Moreover, the IPCC recommended emission factors of 11 and 1% of applied N as NH_3_ to be volatilized and relocated, respectively, to water and soil (assumed equally). Leaching of N_2_O to soil due to runoff was assumed to be 1.1% of the applied N^53^. On the other hand, the PC application on-site does not significantly contribute to greenhouse gasses (GHG); the main GHG emissions arise from water consumption for PC curing^54^. In addition, 22.1 and 12.9% leaching of calcium and silicon ions, respectively, were assumed for plain PC samples compared to non-leached samples (directly after casting)^55^.

### Life cycle impact assessment

The environmental impacts in this study were assessed using the CML-IA methodology^56^. The processes were divided into: external processes, which include transportation and energy consumption, and internal processes, for the manufacturing and application of PC and EICP components. The environmental impact categories included global warming, acidification, eutrophication, abiotic depletion, and marine aquatic eco-toxicity.

### Interpretation

In order to enrich the discussion and facilitate cross-validation with the literature, a sensitivity analysis was performed to investigate two critical issues: (1) potential uncertainty of the used IPCC emissions recommendations. , and (2) the impact of the target UCS on the relative LCA findings.

#### Assessment of emissions uncertainty

In this study, sensitivity analysis was conducted to investigate potential uncertainty in selected parameters, particularly GHG emissions, and urea leaching, by varying their values as per the IPCC recommendations^53^. An additional case that assumes full control of the EICP onsite emissions, i.e., zero emissions, was investigated. Moreover, the individual effect of non-fat milk was analyzed by assuming that waste non-fat milk was used instead of the fresh product. Table 1 summarizes the IPCC recommended ranges of cumulative emissions of nitrous oxide and ammonia.

**Table 1:** Sensitivity analysis scenarios for potential variations of EICP on-site emissions.

Scenario	Urea emission factor	Nitrous oxide (% of N applied)	Ammonium (% of N applied)
Non-fat milk (individual effect)	–	–	–
No emissions	Zero emissions	0.00	0.00
Lowest emissions	Minimum values	0.59	4.12
Baseline	Default values	2.11	21.59
Highest emissions	Maximum values	6.15	51.82

#### Impact of target UCS

Another sensitivity analysis was conducted regarding the variation of UCS. The UCS defined within the FU was 1.5 MPa, however, changing the target UCS is expected to alter the LCA results. This involves varying the cementation content of the treated soil, which changes the constituents and the corresponding impacts^46^. In order to link the findings of this study to the recent MICP literature, the sensitivity analysis included a comparative assessment of EICP and PC treated sand emissions during construction compared to MICP treated sand assuming the same target UCS. The target UCS of the EICP and PC treated sand was varied according to results reported by Almajed et al.^26^, where UCS was tested for EICP treated sand at 0.5, 1.0, and 2.0 urea molarities with 1.5, 3.0, and 6.0 g/L enzyme concentrations, respectively. On the other hand, the cement content that produced similar UCS for PC treated sand was obtained at 9.5, 14.0, and 15.0% cement by weight of dry sand, respectively. The LCA of Deng et al.^46^ was used for the MICP treated sand results after unifying the FU to be consistent with this study for the same target UCS values for EICP and PC treated sand. Table 2 summarizes the target UCS values and the varied constituents.

**Table 2:** Sensitivity analysis scenarios for EICP, PC, and MICP treated sand at various target UCS values.

Target unconfined compressive strength (MPa)	Percentage by dry weight of sand for the different constituents				
	EICP (%)			PC (%)	MICP*
	Urea	CaCl_2_	Enzyme	Cement content	
0.73	0.91	1.82	3.64	9.5	LCA results were directly reported in terms of MPa of UCS from Deng et al.^46^
1.50 (baseline)	1.115	2.23	4.46	14	
2.40	0.046	0.091	0.182	15	

*Adjusted to current FU (treatment of 1500 m^3^ soil volume).

## Results and discussion

Each environmental impact category was individually discussed, followed by the top three contributing processes in each ground stabilization technique. The sensitivity and uncertainty analyses of selected variables were then introduced. Figures and values are reported per the functional unit defined in this study.

### Global warming potential

**Figure 2:**
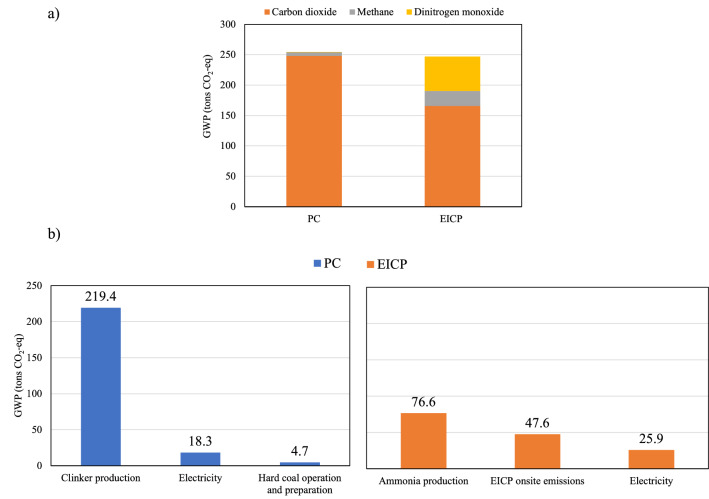
Global warming potential of the PC and EICP soil stabilization techniques: (**a**) total and individual emissions, and (**b**) highest contributing processes.

The contribution of CO_2_, N_2_O, and CH_4_ emissions in the global warming potential (GWP) of each soil stabilization technique is presented in Fig. 2. Carbon dioxide was the highest contributor to GWP in both techniques, accounting for 97 and 70% of the total GWP in the case of using PC and EICP, respectively (Fig. 2a). The CO_2_ emissions were significantly greater than the carbon equivalent of the N_2_O and CH_4_ emissions, although the GWP indices of CH_4_ and N_2_O are 28 and 298 times more than CO_2_, respectively, over a 100-year time horizon. Using PC for soil stabilization resulted in an estimated total GWP of 255 tons of CO_2_-eq; this was reduced to 247 kg CO_2_-eq with the EICP application, i.e., a 3% reduction in GWP. This slight reduction can be partially attributed to utilizing carbon dioxide in urea production plants which results in a positive impact on GWP^57^.

To study the highest contributors to GWP in both techniques, the top three processes that affected GWP were plotted in Fig. 2b. The majority of PC production emissions was from clinker (86%) with 219 tons of CO_2_-eq, followed by electricity usage (7%), and hard coal operation and preparation (1.8%). The reason behind the huge GHG emissions of clinker is the massive amount of energy required to heat the mixture to 1450 °C^58^. On the other hand, the highest GWP contributors from EICP were ammonia production (during urea production) and onsite emissions, with 31 and 20% of the total GWP, respectively. These results are in agreement with Raymond et al.^43^, in which process emissions were found to be the highest contributor, followed by onsite EICP emissions. In addition, in the urea production process generation of ammonia gas (gasification) consumes 60–70% of the total supplied energy^59,60^. Another key GWP contributor was the non-fat milk powder used as an additive to improve the EICP cementation efficiency, with ~ 15% of the total GWP of the EICP process.

### Acidification potential

**Figure 3:**
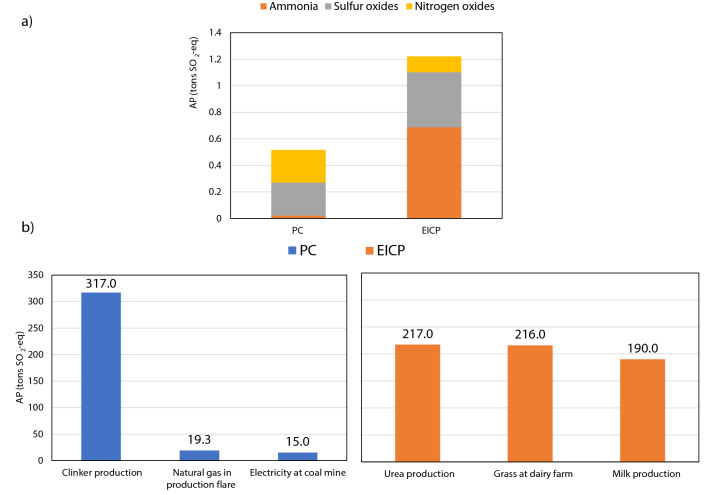
Acidification potential of the PC and EICP soil stabilization techniques: (**a**) total and individual emissions, and (**b**) highest contributing processes.

Acidification potential (AP) is particularly affected by processes involving SO_x_, NH_3,_ and NO_x_ emissions^61^. As shown in Fig. 3a, SO_x_, NH_3,_ and NO_x_ emissions were present with different amounts in both soil stabilization techniques. Although PC production processes were found to largely contribute to AP^63^, the present results revealed that PC had AP of 517 kg SO_2_-eq which is 57.7% less than that of EICP. The major contributor is the ammonia emissions from EICP, which equaled 687.2 kg SO_2_-eq compared to 21.0 kg SO_2_-eq from PC. On the other hand, EICP produced 50% fewer nitrogen oxides and 40% more sulfur oxides compared to PC. The main contributor to AP in PC was from clinker production, with 60% of total AP from PC. As shown in Fig. 3b, urea production emissions, grass planting at dairy farms, and non-fat milk production had 17.8, 17.7, and 15.5%, respectively, of total AP from EICP. Grass at dairy farms had a high impact on AP due to the usage of fertilizers. In addition to its nitrogen oxide and ammonia emissions to the air, grass farming results in direct heavy metal discharges into water ecosystems as stated in Agri-footprint 5.0^62^.

### Eutrophication potential

**Figure 4:**
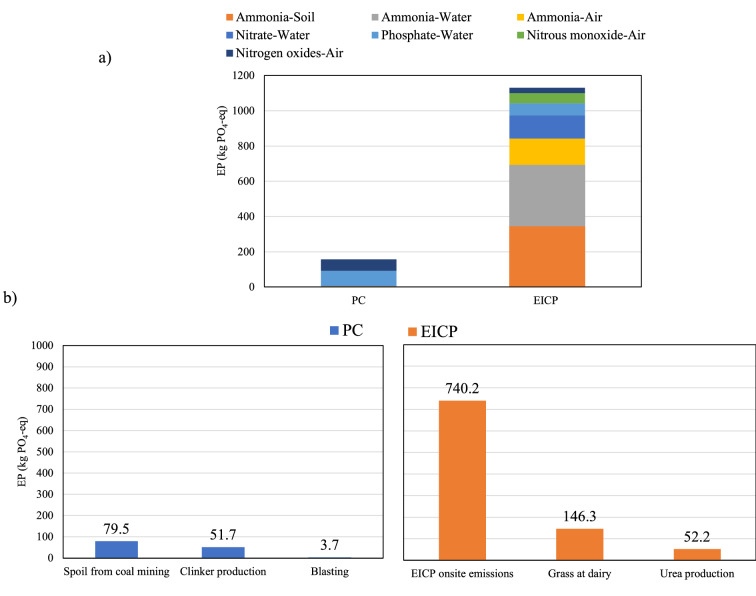
Eutrophication potential of the PC and EICP soil stabilization techniques: (**a**) total and individual emissions, and (**b**) highest contributing processes.

Eutrophication potential (EP) is caused by nutrients loadings of mainly nitrogenous and phosphorus compounds, in soil, water, or air that cause rapid algal growth^63^. As shown in Fig. 4a, the EICP technique had several significant EP-related emissions in water, air, and soil. The total ammonia emissions in water, air, and soil from EICP production and reactions by-product onsite added up to 843 kg-PO_4_^–3^, i.e., 72% of the total EP of EICP. In contrast, PC production and application produced 86% lower EP compared to EICP. This percentage could be decreased by 22.3% by controlling the EICP emissions on-site, as they contribute to around 63.7% of the total EICP EP. On-site EICP emissions were the most significant contributor to EP, which is in line with the findings of Raymond et al.^43^. As shown in Fig. 4b, the emissions of PC production were mostly due to spoils from coal mining with 64% of the total EP of PC. Coal spoils are acidic and contain metal contamination that can leach to ecosystems due to their low water holding capacity^64^.

### Marine aquatic ecotoxicity potential

**Figure 5:**
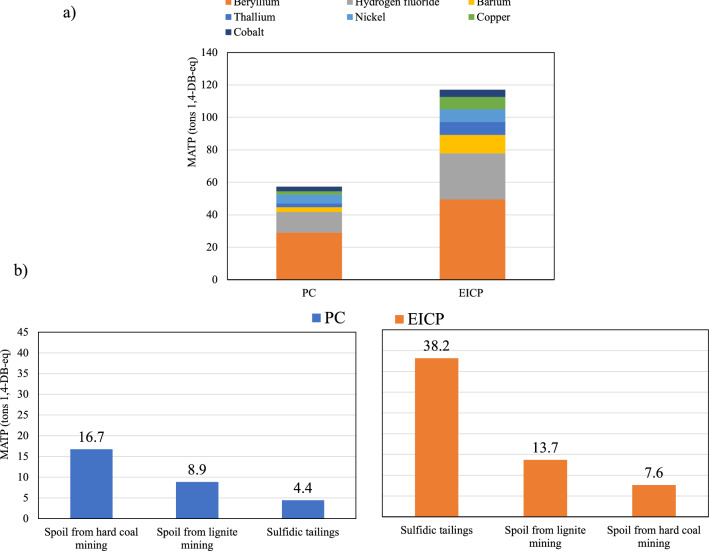
Marine aquatic ecotoxicity potential of the PC and EICP soil stabilization techniques: (**a**) total and individual emissions, and (**b**) highest contributing processes.

Marine aquatic ecotoxicity potential (MAETP) is defined as the impact on organisms in seawater due to toxic substances emitted to ecosystems. As shown in Fig. 5a, the EICP had nearly twice the impact on MAETP compared to PC. Beryllium discharged in water was found to be the highest contributor to MAETP in the EICP and PC techniques with 39.8 and 46.1%, followed by hydrogen fluoride emissions to air (22.7 and 20.2%) of the total, respectively. The main contributing process for such high emissions in EICP was the sulfidic tailings (~ 30.4% of EICP MAETP) resulting from mining sulfidic minerals. The sulfidic tailings are one of the worst environmental impacts to the mining industry and have been considered as the largest environmental liability of the mining industry^65^. On the other hand, similar to EP, the largest contributor to MAETP in PC production was the spoils from coal mining with a total contribution of 26.5%, as shown in Fig. 5b.

### Abiotic depletion potential

**Figure 6:**
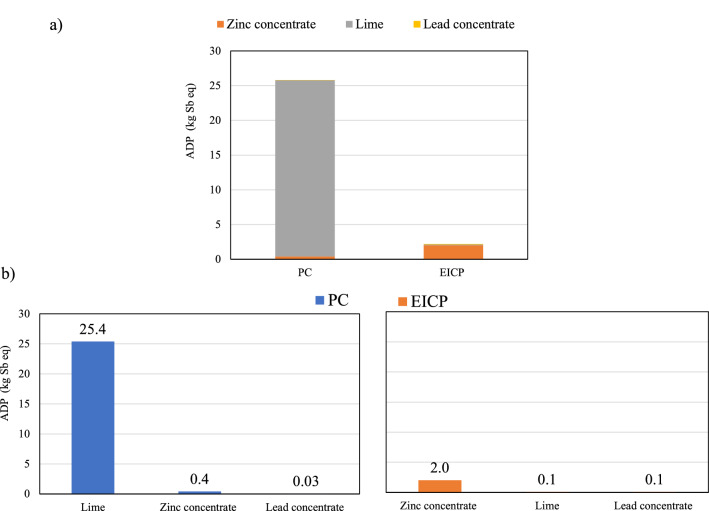
Abiotic depletion potential of resources for the PC and EICP soil stabilization techniques: (**a**) total and individual emissions, and (**b**) highest contributing processes.

Abiotic depletion potential (ADP) is the depletion of resources from non-organic, non-living materials, e.g., air, land, freshwater^66^. As shown in Fig. 6a, EICP outperformed the PC, with nearly 90% less ADP. The most contributing process was the co-production of lime in zinc mine operation, which accounts for 98% of total ADP for PC production as shown in Fig. 6b. Previous studies have discussed the significant amount of lime co-produced with zinc concentrate in mining and beneficiation processes^67^. On the other hand, the zinc concentrate production processes from mining operation resulted in the highest EICP contribution to ADP, where 90% of this ADP was from the production of urea.

### Impacts of external processes

The external processes refer to onsite operations and transportation of final products from local suppliers to site locations. Overall, all materials were available locally near the study area. Using the PC in the soil stabilization involved more transported weights, compared to EICP which was assumed to have longer travel distances. In both cases, the ozone layer depletion potential (ODP) was the most affected environmental impact category, with a 16% higher impact from PC compared to EICP despite shorter distances assumed in the PC case. The next two categories that were severely impacted by external processes were the AP and photochemical ozone creation potential (POCP). PC transportation had a higher impact on AP and POCP by 21.7 and 28.2%, respectively, compared to EICP. The highest difference between PC and EICP was found in GWP, where the external processes of PC produced 71.1% higher GWP compared to those of EICP. The main reason for the lower impact of EICP in overall external processes is the lighter weights of EICP constituents. The total raw materials weight transported in the case of the EICP ground stabilization technique was nearly 1/3 compared to those in the PC case.

### Sensitivity analysis

#### Assessment of emissions uncertainty

**Figure 7:**
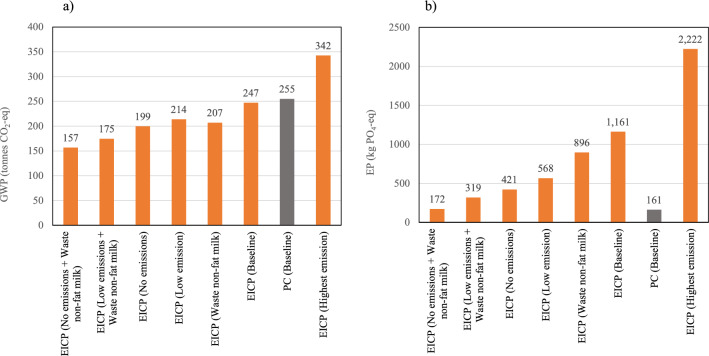
Sensitivity analysis scenarios of EICP compared to the PC baseline scenario for: (**a**) global warming potential, and (**b**) eutrophication potential.

Evaluation of the relative weight of each of the hotspots, i.e., critical processes that have the greatest adverse impact on the environment, in the environmental impact of the EICP process. As shown in Fig. 7, the most affected environmental impact categories by those changes were the GWP and EP, respectively. The GWP has decreased by 13.4% when the lowest emissions were assumed, whereas applying the highest emissions increased the overall GWP by 38.5% compared to baseline, as shown in Fig. 7a. The high GWP in the case of the highest EICP emissions would be 34.2% higher than that of the PC technique. In the no emissions and waste non-fat milk scenario, the GWP decreased by 38.5% compared to the PC scenario. Furthermore, EP has significantly increased in the highest emission scenario (Fig. 7b); it nearly doubled the EICP baseline scenario, which is 7.2-fold greater than the case of PC-treated soils. In contrast, in the no emissions scenario adopting waste non-fat milk, the EICP impacts on EP would decrease to be similar to that of PC. Overall, the sensitivity analysis corroborates the high effect of EICP onsite emissions and non-fat milk on the GWP and EP; reducing those emissions would favor EICP over PC. In terms of AP, using waste non-fat milk would reduce the AP of EICP by 38.1%, which will make the EICP 49% higher than PC in AP compared to 140% higher in the case of using fresh non-fat milk. The individual contribution of non-fat milk was identified to be 39 tons CO_2_-eq in GWP, 249 kg PO_4_^–3^-eq in EP, and 465 kg SO_2_-eq in AP.

#### Impact of target UCS

**Figure 8:**
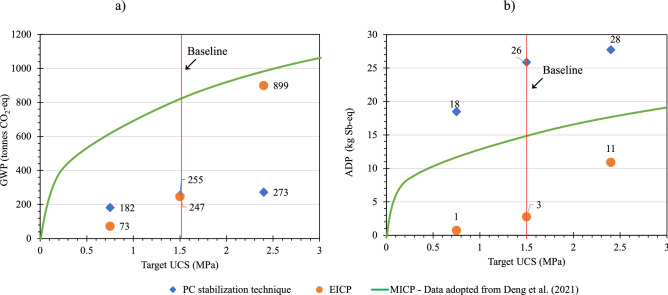
Sensitivity analysis scenarios of EICP compared to PC and MICP for: (**a**) global warming potential, and (**b**) abiotic depletion potential.

Different sensitivity analysis scenarios were proposed to evaluate the effect of target UCS on the relative findings of this LCA study. The effect of target UCS change was assessed by comparing the effect on GWP and ADP between EICP, PC, and MICP stabilization techniques. The effects of varying target UCS at different levels of 0.73, 1.5, and 2.4 MPa on the GWP and ADP are summarized in Fig. 8a,b, respectively. Generally, the MICP soil stabilization technique had higher GWP compared to EICP and PC stabilization techniques at different UCS targets, as shown in Fig. 8a. The GWP has increased by 263.9, 7.1, and 37.5%, when the target UCS was increased from 1.5 to 2.4 MPa for EICP, PC, and MICP stabilization techniques respectively. On the other hand, decreasing the target UCS from 1.5 to 0.73 MPa resulted in the reduction of the GWP by 238.4, 40.1, and 33.3%, respectively. This suggests that the EICP soil stabilization technique is potentially a better environmental option, in terms of its carbon footprint, when the UCS of the treated soil is equal to or less than 1.5 MPa, whereas increasing the UCS above 1.5 MPa exponentially increased the GWP of EICP, making it a much less favorable alternative to PC and slightly better than MICP.

The ADP of the MICP stabilization technique was found to be lower than cement and higher than EICP (Fig. 8b). At a target UCS of 1.5 MPa, MICP consumed 73.3% less non-renewable resources compared to PC. Increasing the UCS from 1.5 to 2.4 MPa increased the ADP of EICP, PC, and MICP stabilization techniques by 266.7, 13.3, and 7.7%, respectively. Similar to GWP, the ADP of EICP-treated soil exponentially increased at higher target UCS, unlike MICP and PC. Overall, the high GWP and ADP of MICP compared to EICP can be attributed to the different chemicals required to maintain the bacterial activity in MICP; each liter of the bacterial culture medium contains, 10 g of (NH_4_)_2_SO_4_, 20 g of yeast extract, and 10 mol of NiCl_2_. In addition, to maintain a suitable pH value for bacterial growth in the range of 8.5–9, sodium hydroxide, which typically causes significant negative environmental impacts, is used. On the other hand, Deng et al.^46^ assumed that MICP has no on-site emissions as all CO_2_ generated by urea hydrolysis changes to CO_3_^–2^.

### Limitations and recommendations

Similar to all LCA studies, the findings of this assessment have to be carefully interpreted taking into consideration the various assumptions and project-specific conditions. To enable proper cross-comparison of the study outcomes, the following limitations and considerations must be accounted for:

- Different FU would significantly change the proportions of constituents required for both techniques. Moreover, the soil gradation and source were reported to affect the strength and performance of both PC and EICP treated soils^26^. Therefore, the current study results are limited to the constituents of both PC and EICP, and the sand soil reported in this study thus, more studies are required to investigate the effect of soil type on the results of LCA.
- The lifecycle inventory analysis was computed in this study considering the sub-tropical arid climate in Dubai. Changing the project geographic location would vary certain inputs, e.g., emission factors, possibly leading to different outcomes.
- The scarcity of data in the literature, particularly those related to the field onsite emissions of EICP, has led to adopting the IPCC recommendations. The uncertainty analysis of EICP emissions showed a substantial impact on GWP and EP. To avoid this critical assumption, laboratory and field measurements for those emissions and their leaching rates are highly recommended for future studies.
- Although the amounts of EICP constituents were significantly less than PC, the costs associated with the implementation of EICP would be substantially higher than those of the conventional PC technique. This unmatched benefit of PC is due to the numerous technological enhancements and cost optimization achieved in the cement industry for decades. As the EICP soil stabilization technique becomes gradually commercialized, its economic performance would eventually improve compared to PC.

Based on the environmental hotspots indicated in this study, it is clear that the overall performance of the EICP soil stabilization technique can be significantly improved by applying sustainability and mitigation measures to reduce the emissions of selected sub-processes, as follows:

- Urea production was one of the key environmental hotspots in the EICP technique. Several techniques could decrease the energy consumed by the gasification process in urea production, e.g., heat recovery of primary reformer in the natural gas reforming process^68^ which employs highly efficient catalysts to reduce steam use in gasification. Recovery of urea from fresh mammals’ urine can be also investigated^69^.
- The contribution of onsite emissions was quite high in the overall EICP performance. This is because of high levels of nitrogen dioxide and ammonia gas produced during the hydrolysis process of urea. Studies in the field of reducing nitrous oxide and ammonia emission from the EICP soil stabilization technique are scarce. Cheng et al.^20^ investigated the atmospheric ammonia produced from “low-pH treated” MICP, which is a hydrolysis process similar to EICP. A 90% reduction of ammonia was achieved by reducing the initial pH of the solution which holds the ammonia ions in liquid form.
- The onsite emissions were found to be the second contributor to the GWP in the case of EICP treated soils. Several studies had focused on the removal of ammonium from soils. For example, Wang et al.^70^ have shown that using electro-kinetics was effective in electricizing ammonia in soils which significantly reduces the EP. However, the electricizing technique still needs additional studies on large-scale field studies to improve its applicability.
- The non-fat milk powder contributed to ~ 15% of the total GWP from the EICP processes. GWP due to emissions from grass farming and milk production varies significantly depending on the production processes. Reduction of GWP in dairy farms could be achieved by adopting other production strategies or sustainable manure management techniques, such as anaerobic digestion that could reduce the GWP of dairy farms up to 25% compared to conventional techniques^61^. The use of expired milk as a raw material in the EICP cementing may reduce the cost and improve the environmental sustainability of the EICP technique. From another perspective, using non-fat milk in EICP can be fully eliminated. Cui et al.^27^ achieved ~ 1.5 MPa compressive strength of EICP treated specimens (similar to the present FU) without using milk, however, in order to achieve such strength, the researchers applied three cycles of the EICP solution.
- In the EICP technique, four components are manufactured in different factories and agro-industrial processes, which is not the case with centralized cement production. Having multiple entities producing various components to be combined into one product would reduce the overall environmental efficiency of the system^71^; this is on top of the additional transportation-related environmental burdens.
- The potentially improved performance of EICP treated soils has not been considered in this study. EICP treated soils were proven to perform well under certain conditions such as sulfate contamination^25^. Comparative laboratory experiments on EICP and PC-treated soils under harsh environmental conditions, e.g., heavy metal leaching, freeze and thaw cycles, wetting and drying cycles, and sulfate contamination, are required to assess the effect of durability on the LCA analysis.
- To date, the EICP and MICP research had been mostly conducted on small-scale experiments and using laboratory-grade materials. Moving towards the development of the EICP/MICP field techniques, it is expected that larger-scale applications with industrial-grade materials would improve the overall environmental performance of both emerging methods compared to the well-established PC.

## Conclusion

This study presents a comparative LCA to evaluate the use of EICP for soil stabilization compared to PC used for soil stabilization. PC is well known for its rapid and reliable performance in stabilizing sand mechanical behavior, whereas EICP is being extensively investigated as a sustainable material that would replace conventional stabilization techniques. The results revealed PC had eightfold higher ADP and 3% higher GWP compared to the EICP soil stabilization technique. While the PC has nearly 50% MAETP and AP compared to EICP; due to high onsite emissions and ammonia produced in the urea production. On the other hand, the sensitivity analysis for EICP scenarios has shown that onsite emissions during the application of EICP have the highest impact on GWP and EP. Controlling EICP emissions and adopting waste non-fat milk would result in reducing EICP GWP to 37% from cement GWP. In addition, using waste non-fat milk and controlling EICP emissions reduces the EP of EICP from 621 to 103% compared to Portland cement soil stabilization technique EP. In addition, another sensitivity analysis was conducted to investigate the effect of treated soil’s UCS on its environmental performance. The results suggested that, at lower UCS values, the EICP treated soils achieved less carbon footprint compared to PC and MICP treated soils.

## Supplementary Information

Supplementary Information.
