# Supplementary material for: Life cycle assessment of biocemented sands using enzyme induced carbonate precipitation (EICP) for soil stabilization applications
Source: Sci Rep. 2022 Apr 11;12:6032. doi: 10.1038/s41598-022-09723-7 (PMC9001663; doi:10.1038/s41598-022-09723-7)
Supplement: Supplementary file 1 — Supplementary Information. [file 41598_2022_9723_MOESM1_ESM.docx]

Appendix 1

The urea production flow adopted in this study is discussed in Ecoinvent database (Ruiz et al. 2020). Urea or carbamide is an amide compound with chemical formula (NH_2_)_2_CO. It plays an important role in mammals’ metabolism and has various industrial applications. A schematic of a urea production process is illustrated in Fig. 1. The basic urea process consists of two main reactions shown in Equations (1) and (2):

| 2 NH_3_ + CO_2_ ⇌ H_2_N-COONH_4_ΔH 298 =−58.87 kJ/mol (Carbamate formation) | (1) |
| --- | --- |
| H_2_N-COONH_4_ ⇌  (NH_2_)_2_CO + H_2_O ΔH 298 =−56.46 kJ/mol (Urea conversion) | (2) |

The first reaction is fast exothermic and happens at high temperature and high pressure to produce ammonium carbamate (H_2_N-COONH_4_). This component is decomposed through a slow endothermic reaction in the second stage to form ammonia. The detailed reactions in all stages of the production are mentioned at the end of this appendix.

The typical production flow of urea is shown in Fig. 1. The summarized urea production flow used in this study is shown in Fig. 2 exported from ecoinvent (Moreno et al. 2019).

Generally, for producing one tonne of urea, 0.735–0.750 t CO_2_ is consumed (Muradov, 2014). One of the key applications of urea is in fertilizers where it reacts with water, releasing CO_2_ and ammonia to the soil. Furthermore, the amount of CO_2_ emissions in the urea production process is 2.27 tonnes of CO_2_-eq. per tonne of CO_2_ utilized (Brinckerhoff, 2011). Consequently, the concept of CO_2_ utilization in urea production is not recognized as a carbon reduction measure (Muradov, 2014) which was adopted in this study following ecoinvent database (Ruiz et al. 2020).

The production of 1 kg of urea following the discussed method produces massive number of by-products into the water, soil, and air. The total number of main and sub process are 14693 according to ecoinvent utilization in Simapro. Most 30 affecting parameters are summarized in Table 1 exported from Simapro for production of 1 kg of urea. The detailed description of each stage is followed after Table 1.


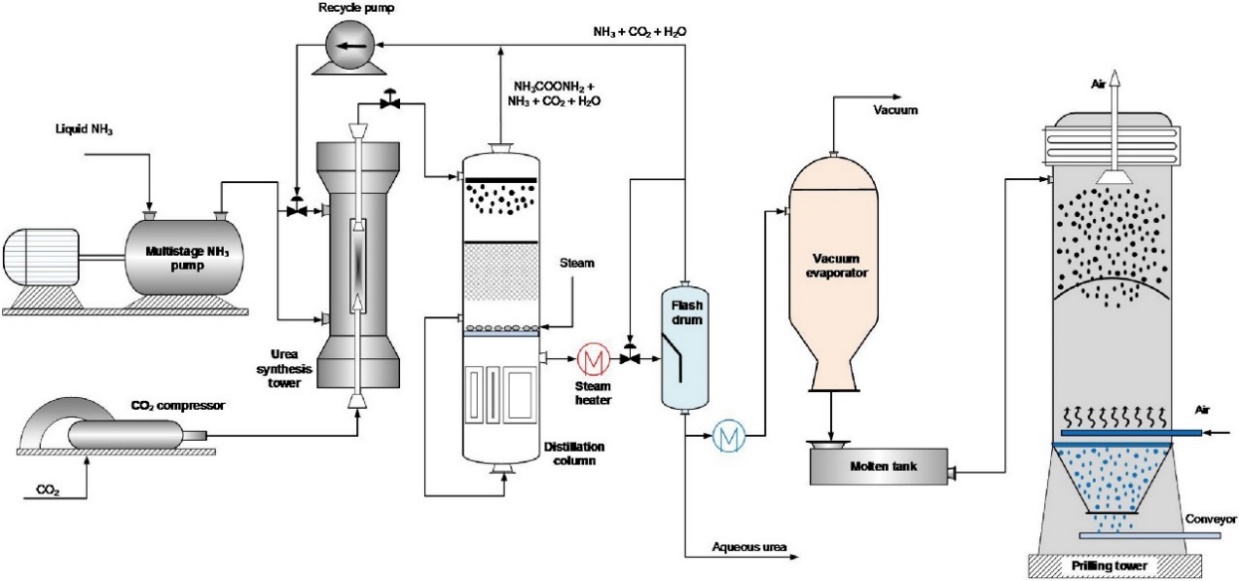


Fig 1. Typical conventional urea production plant (Exported from Rafiee et al. 2019)


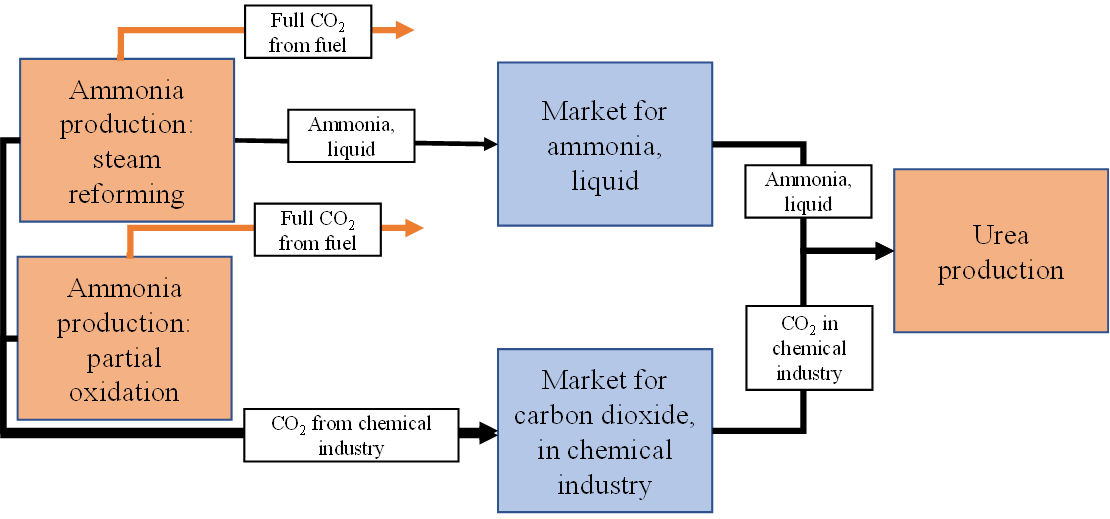


Fig. 2: Adopted urea production flow in Ecoinvent 3.7 database

References:

Brinckerhoff, P. (2011). Accelerating the uptake of CCS: industrial use of captured carbon dioxide. *Global CCS Institute*, *260*.

Rafiee, A., Khalilpour, K. R., & Milani, D. (2019). CO2 conversion and utilization pathways. In Polygeneration with Polystorage for Chemical and Energy Hubs (pp. 213-245). Academic Press.

Moreno Ruiz, E., Valsasina, L., Fitzgerald, D., Brunner, F., Symeonidis, A., Bourgault, G., & Wernet, G. (2019). Documentation of changes implemented in the Ecoinvent database v3. 6. Ecoinvent Association, Zürich, Switzerland.

Moreno Ruiz, E., Valsasina, L., FitzGerald, D., Symeonidis, A., Turner, D., Müller, J., ... & Wernet, G. (2020). Documentation of changes implemented in ecoinvent database v3. 7 & v3. 7.1. ecoinvent Association. *Zürich, Switzerland*.

Muradov, N. (2014). Industrial Utilization of CO 2: A Win–Win Solution. In *Liberating Energy from Carbon: Introduction to Decarbonization* (pp. 325-383). Springer, New York, NY.

Table 1. Most affecting parameters for producing 1 kg of urea (exported from Simapro software following Ecoinvent database)

| No | Process | Unit | Required amount to obtain 1 kg of urea |
| --- | --- | --- | --- |
| 1 | Total water used | m^3^ | 6.53153962 |
| 2 | Water, RoW | m^3^ | 3.872522791 |
| 3 | Gas, natural/m3 | m^3^ | 0.870148009 |
| 4 | Radon-222 | Bq | 39642.868 |
| 5 | Noble gases, radioactive, unspecified | Bq | 9909.831 |
| 6 | Hydrogen-3, Tritium | Bq | 1265.8423 |
| 7 | Xenon-133 | Bq | 35.769845 |
| 8 | Radioactive species, other beta emitters | Bq | 32.025582 |
| 9 | Hydrogen-3, Tritium | Bq | 20.448217 |
| 10 | Xenon-135 | Bq | 12.207114 |
| 11 | Carbon-14 | Bq | 10.303005 |
| 12 | Radium-226 | Bq | 5.6577107 |
| 13 | Xenon-135m | Bq | 4.8139607 |
| 14 | Thorium-228 | Bq | 4.0234613 |
| 15 | Radium-228 | Bq | 3.6717515 |
| 16 | Radon-220 | Bq | 1.5292543 |
| 17 | Radioactive species, Nuclides, unspecified | Bq | 1.2992353 |
| 18 | Xenon-138 | Bq | 1.1316678 |
| 19 | Radium-224 | Bq | 1.0058648 |
| 20 | Thorium-230 | Bq | 0.72267094 |
| 21 | Krypton-85m | Bq | 0.71544358 |
| 22 | Xenon-131m | Bq | 0.52445453 |
| 23 | Strontium-90 | Bq | 0.48344162 |
| 24 | Polonium-210 | Bq | 0.36644578 |
| 25 | Uranium alpha | Bq | 0.33322535 |
| 26 | Lead-210 | Bq | 0.28247689 |
| 27 | Krypton-85 | Bq | 0.26525137 |
| 28 | Lead-210 | Bq | 0.20761849 |
| 29 | Cesium-137 | Bq | 0.20486507 |
| 30 | Niobium-95 | Bq | 0.16440164 |

This dataset was already contained in the ecoinvent database version 2. It was not individually updated during the transfer to ecoinvent version 3. Life Cycle Impact Assessment results may still have changed, as they are affected by changes in the supply chain, i.e. in other datasets. This dataset was generated following the ecoinvent quality guidelines for version 2. It may have been subject to central changes described in the ecoinvent version 3 change report (http://www.ecoinvent.org/database/ecoinvent-version-3/reports-of-changes/), and the results of the central updates were reviewed extensively. The changes added e.g. consistent water flows and other information throughout the database. The documentation of this dataset can be found in the ecoinvent reports of version 2, which are still available via the ecoinvent website. The change report linked above covers all central changes that were made during the conversion process.]

This dataset represents the production of urea-compounds (1 kg of active ingredient). Raw materials are modelled with a stoichiometric calculation. Energy consumption is modelled with data from a similar process. The emissions are estimated. Infrastructure is included with a default value.

Production volume: 4900020.35195531 kg

Included activities start: From reception of precursors (acetic anhydride, acetonitrile, acrylonitrile, aluminium, ammonia, ammonium chloride, calcium carbide, carbon dioxide, chlorine, cumene, dichloromethane, dimethylamine, dimethylsulfate, ethanol, hydrochloric acid, hydrogen, hydrogen cyanide, hydrogen fluoride, hydrogen sulfide, natural gas, nitrogen, oxygen, methanol, nitric acid, o-dichlorobenzene, p-dichlorobenzene, phosgene, 1-propanol, propylene, sodium chloride, sodium hydroxide, sulfur, sulfuric acid, sulfur dioxide, sulfur trioxide, toluene, water decarbonised) at the factory gate.

Included activities end: This activity ends with (sulfonyl)urea-compounds at the factory gate. The dataset includes the input materials, energy uses, infrastructure and emissions. The dataset doesn't include emulsives, additives etc.

Geography: The inventory is modelled for rest of the world

Technology level: 3

Technology: [Sulfonyl ] urea are herbicidal compounds with a urea functional group (urea) or a urea functional group having a sulfonyl substituent (sulfonyl urea). Urea are produced by phosgenation of an amine with an amination of the resulting isocyanate. Sulfonylureas are usually produced by reacting a sulfonamide with an amine, or a sulfonamide isocyanate with an amine, or a sulfonamide carbamate with an amine.

The dataset [sulfonyl]urea-compounds was calculated from the arithmetic mean of all inputs and outputs of the production of amidosulfuron, chlortoluron, diuron, isoproterenol, lufenuron, metsulfuron-methyl, nicosulfuron, and thifensulfuron-methyl.

Amidosulfuron is produced in five steps from methane shown in Equations (3) to (7):

| CH_4_ + SO_3_ → CH_4_SO_3_ | (3) |
| --- | --- |
| CH_4_SO_3_ + SOCl_2_ → CH_3_ClO_2_S + SO_2_ + HCl | (4) |
| CH_3_ClO_2_S + CH_5_N → C_2_H_7_NSO_2_ + HCl | (5) |
| C_2_H_7_NSO_2_ + CClNO_3_S → C_3_H_6_N_2_S_2_O_5_ + HCl | (6) |
| C_3_H_6_N_2_O_5_S_2_ + C_6_H_9_N_3_O_2_ → C_9_H_15_N_5_O_7_S_2_ | (7) |

Precursors that are used for the amidosulfuron production are amino-_4_,_6_-dimethoxy pyrimidine (C_6_H_9_N_3_O_2_), chlorosulfonyl isocyanate sulfonamide (CClNO_3_S), dimethyl malonate (C_5_H_8_O_4_), and thionyl chloride (SOCl_2_).

Chlorotoluron is produced in five steps from toluene shown in Equations (8) to (12):

| C_7_H_8_ + HNO_3_ → C_7_H_7_NO_2_ + H_2_O | (8) |
| --- | --- |
| C_7_H_7_NO_2_ + Cl_2_ → C_7_H_6_ClNO_2_ + HCl | (9) |
| C_7_H_6_ClNO_2_ + _3_ H_2_ → C_7_H_8_ClN + _2_ H_2_O | (10) |
| C_7_H_8_ClN + COCl_2_ → C_8_H_6_ClNO + _2_ HCl | (11) |
| C_8_H_6_ClNO + C_2_H_7_N → C_10_H_14_ClN_2_O | (12) |

Diuron is produced in four steps from dichlorobenzene shown in Equations (13) to (16):

| C_6_H_4_Cl_2_ + HNO_3_ → C_6_H_3_Cl_2_NO_2_ + H_2_O | (13) |
| --- | --- |
| C_6_H_3_Cl_2_NO_2_ + _3_ H_2_ → C_6_H_5_Cl_2_N + _2_ H_2_O | (14) |
| C_6_H_5_Cl_2_N + COCl_2_ → C_7_H_3_Cl_2_ON + _2_ HCl | (15) |
| C_7_H_3_Cl_2_NO + C_2_H_7_N → C_9_H_10_Cl_2_N_2_O | (16) |

Isoproturon is produced in four steps from cumene shown in Equations (17) to (20):

| C_9_H_12_ + HNO_3_ → C_9_H_11_NO_2_ + H_2_O | (17) |
| --- | --- |
| C_9_H_11_NO_2_ + _3_ H_2_ → C_9_H_14_N + _2_ H_2_O | (18) |
| C_9_H_14_N + COCl_2_ → C_10_H_11_NO + _2_ HCl | (19) |
| C_10_H_11_NO + C_2_H_7_N → C_12_H_18_N_2_O | (20) |

Lufenuron is produced in five steps from _1_,_4_-dichlorobenzene shown in Equations (21) to (25):

| C_6_H_4_Cl_2_ + Cl_2_ → C_6_H_3_Cl_3_ + HCl | (21) |
| --- | --- |
| C_6_H_3_Cl_3_ + C_3_H_2_F_6_O → C_9_H_4_Cl_2_F_6_O + HCl | (22) |
| C_9_H_4_Cl_2_F_6_O + HNO_3_ → C_9_H_3_Cl_2_F_6_NO_3_ + H_2_O | (23) |
| C_9_H_3_Cl_2_F_6_NO_3_ + _3_ H_2_ → C_9_H_5_Cl_2_F_6_NO + _2_ H_2_O | (24) |
| C_9_H_5_Cl_2_F_6_NO + C_8_H_3_F_2_NO_2_ → C_17_H_8_Cl_2_F_8_N_2_O_3_ | (25) |

Precursors that are used for the lufenuron production are _2_,_4_-difluorobenzyl isocyanate (C_8_H_3_F_2_NO_2_) and _1_,_1_,_2_,_3_,_3_,_3_-hexafluoropropanol (C_3_H_2_F_6_O).

Metsulfuron-methyl is produced in seven steps from sulfur trioxide shown in Equations (26) to (32):

| SO_3_ + HCl → ClSO_2_OH | (26) |
| --- | --- |
| HSO_3_Cl + C_7_H_8_ → C_7_H_7_ClO_2_S + H_2_O | (27) |
| C_7_H_7_ClO_2_S + NH_3_ → C_7_H_9_NO_2_S + HCl | (28) |
| C_7_H_9_NO_2_S + H_2_O → C_7_H_5_NO_3_S + _3_ H_2_ | (29) |
| C_7_H_5_NO_3_S + CH_4_O → C_8_H_9_NO_4_S | (30) |
| C_8_H_9_NO_4_S + C_3_H_5_ClO_2_ → C_11_H_14_NO_6_S + HCl | (31) |
| C_11_H_14_NO_6_S + C_5_H_8_N_4_O → C_14_H_15_N_5_O_6_S + C_2_H_6_O | (32) |

Precursors that are used for the metsulfuron-methyl production are _2_-amino-_4_-methoxy-_6_-methyl-_1_,_3_,_5_-triazine (C_5_H_8_N_4_O), cyanamide (CH_2_N_2_), ethyl-chloro formate (C_3_H_5_ClO_2_), and o-methyl-isourea (C_2_H_6_N_2_O).

Nicosulfuron is produced in eleven steps from propene shown in Equations (33) to (43):

| C_3_H_6_ + O_2_ → C_3_H_4_O + H_2_O | (33) |
| --- | --- |
| 2C_3_H_4_O + NH_3_ → C_6_H_7_N + 2H_2_O | (34) |
| C_6_H_7_N + 4Cl_2_ → C_6_H_3_Cl_4_N + 4HCl | (35) |
| C_6_H_3_Cl_4_N + _2_ H_2_O → C_6_H_4_ClNO_2_ + 3HCl | (36) |
| C_6_H_4_ClNO_2_ + H_2_S → C_6_H_5_NO_2_S + HCl | (37) |
| C_6_H_5_NO_2_S + CH_4_O → C_7_H_7_NO_2_S + H_2_O | (38) |
| C_7_H_7_NO_2_S + 2H_2_O + 3Cl_2_ → C_7_H_6_ClNO_4_S + 5 HCl | (39) |
| C_7_H_6_ClNO_4_S + C_4_H_11_N → C_11_H_16_N_2_O_4_S + HCl | (40) |
| C_11_H_16_N_2_O_4_S + C_4_H_12_NAl → C_12_H_19_N_3_O_3_S + C_3_H_9_OAl | (41) |
| C_12_H_19_N_3_O_3_S + C_2_HF_3_O_2_ → C_8_H_11_N_3_O_3_S + C_6_H_9_F_3_O_2_ | (42) |
| C_8_H_11_N_3_O_3_S + C_7_H_7_N_3_O_3_ → C_15_H_18_N_6_O_6_S | (43) |

Precursors that are used for the nicosulfuron production are tert-butyl amine (C_4_H_11_N), (N,N-dimethyl amino)-dimethyl aluminium (C_4_H_12_NAl), dimethyl malonate (C_5_H_8_O_4_), _2_-isocyanato-_4_,_6_-dimethoxy pyrimidine (C_7_H_7_N_3_O_3_), sodium (Na), and trifluoroacetic acid (C_2_HF_3_O_2_).

Thifensulfuron-methyl is produced in nine steps from acetic acid shown in Equations (44) to (52):

| C_2_H_4_O_2_ + Cl_2_ → C_2_H_3_ClO_2_ + HCl | (44) |
| --- | --- |
| C_2_H_3_ClO_2_ + NaSH → C_2_H_4_O_2_S + NaCl | (45) |
| C_2_H_4_O_2_S + CH_4_O → C_3_H_6_O_2_S + H_2_O | (46) |
| C_3_H_6_O_2_S + CH_2_ClN_3_ → C_6_H_7_NO_2_S + HCl | (47) |
| C_6_H_7_NO_2_S + _2_ NaNO_2_ + HCl → C_6_H_5_ClN_2_O_2_S + 2 H_2_O + NaCl | (48) |
| C_6_H_5_ClN_2_O_2_S + SO_2_ → C_6_H_5_ClO_4_S_2_ + N_2_ | (49) |
| C_6_H_5_ClO_4_S_2_ + NH_3_ → C_6_H_7_NO_4_S_2_ + HCl | (50) |
| C_6_H_7_NO_4_S_2_ + COCl_2_ → C_7_H_5_NO_5_S_2_ + 2 HCl | (51) |
| C_7_H_5_NO_5_S_2_ + C_5_H_8_N_4_O → C_12_H_14_N_5_O_6_S_2_ | (52) |

Precursors that are used for the thifensulfuron-methyl production are _2_-amino-_4_-methoxy-_6_-methyl-_1_,_3_,_5_-triazine (C_5_H_8_N_4_O), chloroacrylonitrile (CH_2_ClN_3_), cyanamide (CH_2_N_2_), sodium hydrogensulfide (NaSH), and sodium nitrite (NaNO_2_).

Precursors :

Amino-_4_,_6_-dimethoxy pyrimidine (C_6_H_9_N_3_O_2_) is produced in five steps from calcium carbide shown in Equations (53) to (57):

| CaC_2_ + N_2_ → CaCN_2_ + C | (53) |
| --- | --- |
| CaCN_2_ + CO_2_ + H_2_O → CH_2_N_2_ + CaCO_3_ | (54) |
| CH_2_N_2_ → C_2_H_4_N_4_ | (55) |
| C_2_H_4_N_4_ + 2 NH_4_Cl → CH_5_N_3_ + 2 HCl | (56) |
| CH_5_N_3_ + C_5_H_8_O_4_ → C_6_H_9_N_3_O_2_ + 2 H_2_O | (57) |

_2_-Amino-_4_-methoxy-_6_-methyl-_1_,_3_,_5_-triazine (C_5_H_8_N_4_O) is produced in three steps from acetonitrile shown in Equations (58) to (60):

| C_2_H_3_N + CH_4_O → C_3_H_7_NO | (58) |
| --- | --- |
| C_3_H_7_NO + CH_2_N_2_ → C_4_H_6_N_2_O + NH_3_ | (59) |
| C_4_H_6_N_2_O + C_2_H_6_N_2_O → C_5_H_8_N_4_O + CH_4_O | (60) |

tert-Butyl amine (C_4_H_11_N) is produced in two steps from _2_-methylpropene shown in Equations (61) to (62):

| C_4_H_8_ + H_2_O + HCN + H_2_SO_4_ → C_5_H_11_NO + H_2_SO_4_ | (61) |
| --- | --- |
| C_5_H_11_NO + H_2_O + H_2_SO_4_ + 3 NaOH → C_4_H_11_N + NaCHO_2_ + Na_2_SO_4_ + 3 H_2_O | (62) |

Chloroacrylonitrile (CH_2_ClN_3_) is produced from acrylonitrile and chlorine shown in Equation (63):

| C_3_H_3_N + Cl_2_ → CH_2_ClN_3_ + HCl | (63) |
| --- | --- |

Chlorosulfonyl isocyanate sulfonamide (CClNO_3_S) is produced in three steps from hydrogen chloride and sulfur trioxide shown in Equations (64) to (66):

| HCl + SO_3_ → ClSO_2_OH | (64) |
| --- | --- |
| HSO_3_Cl + NH_3_ → H_2_SNO_2_Cl + H_2_O | (65) |
| H_2_SNO_2_Cl + COCl_2_ → CClNO_3_S + 2 HCl | (66) |

Cyanamide (CH_2_N_2_) is produced in two steps from calcium carbide shown in Equations (67) and (68):

| CaC_2_ + N_2_ → CaCN_2_ + C | (67) |
| --- | --- |
| CaCN_2_ + CO_2_ + H_2_O → CH_2_N_2_ + CaCO_3_ | (68) |

_2_,_4_-Difluorobenzyl isocyanate (C_8_H_3_F_2_NO_2_) is produced in five steps from toluene shown in Equations (69) to (73):

| C_7_H_8_ + 2 Cl_2_ → C_7_H_6_Cl_2_ + 2 HCl | (69) |
| --- | --- |
| C_7_H_6_Cl_2_ + _2_ HF → C_7_H_6_F_2_ + 2 HCl | (70) |
| C_7_H_6_F_2_ + HNO_3_ → C_7_H_4_F_2_O_2_ + H_2_O | (71) |
| C_7_H_4_F_2_O_2_ + NH_3_ → C_7_H_5_F_2_NO + H_2_O | (72) |
| C_7_H_5_F_2_NO + COCl_2_ → C_8_H_3_F_2_NO_2_ + 2 HCl | (73) |

(N,N-Dimethyl amino)-dimethyl aluminium (C_4_H_12_NAl) is produced in three steps from methylchloride shown in Equations (74) to (76):

| 3 CH_3_Cl + 2 Al → C_3_H_9_Al_2_Cl_3_ | (74) |
| --- | --- |
| C_3_H_9_Al_2_Cl_3_ + 3 CH_3_Cl + 6 Na → C_6_H_18_Al_2_ + 6 NaCl | (75) |
| C_6_H_18_Al_2_ + 2 C_2_H_7_N → 2 C_4_H_12_NAl + 2 CH_4_ | (76) |

Dimethyl malonate (C_5_H_8_O_4_) is produced in four steps from acetic acid shown in Equations (77) to (80):

| C_2_H_4_O_2_ + Cl_2_ → C_2_H_3_ClO_2_ + HCl | (77) |
| --- | --- |
| C_2_H_3_ClO_2_ + HCN → C_3_H_3_NO_2_ + HCl | (78) |
| C_3_H_3_NO_2_ + NaOH → NaC_3_H_2_NO_2_ + H_2_O | (79) |
| NaC_3_H_2_NO_2_ + 2 CH_4_O + HCl → C_5_H_8_O_4_ + NH_3_ + NaCl | (80) |

Ethyl chloroformate (C_3_H_5_ClO_2_) is produced from ethanol and phosgene shown in Equation (81):

| C_2_H_6_O + CCl_2_O → C_3_H_5_ClO_2_ + HCl | (81) |
| --- | --- |

_1_,_1_,_2_,_3_,_3_,_3_-Hexafluoropropanol (C_3_H_2_F_6_O) is produced in two steps from propanol shown in Equations (82) and (83):

| C_3_H_8_O + 6 Cl_2_ → C_3_H_2_Cl_6_O + 6 HCl | (82) |
| --- | --- |
| C_3_H_2_Cl_6_O + 6 HF → C_3_H_2_F_6_O + 6 HCl | (83) |

_2_-Isocyanato-_4_,_6_-dimethoxy pyrimidine (C_7_H_7_N_3_O_3_) is produced in two steps from calcium carbide shown in Equations (84) to (89):

| CaC_2_ + N_2_ → CaCN_2_ + C | (84) |
| --- | --- |
| CaCN_2_ + CO_2_ + H_2_O → CH_2_N_2_ + CaCO_3_ | (85) |
| CH_2_N_2_ → C_2_H_4_N_4_ | (86) |
| C_2_H_4_N_4_ + _2_ NH_4_Cl → CH_5_N_3_ + 2 HCl | (87) |
| CH_5_N_3_ + C_5_H_8_O_4_ → C_6_H_9_N_3_O_2_ + 2 H_2_O | (88) |
| C_6_H_9_N_3_O_2_ + COCl_2_ → C_7_H_7_N_3_O_3_ + 2 HCl | (89) |

o-Methyl-isourea (C_2_H_6_N_2_O) is produced from cyanamide and methanol shown in Equation (90):

| CH_2_N_2_ + CH_4_O → C_2_H_6_N_2_O | (90) |
| --- | --- |

Sodium (Na) is produced by electrolysis of sodium chloride shown in Equation (91):

| 2 NaCl → 2 Na + Cl_2_ | (91) |
| --- | --- |

Sodium hydrogen sulfide (NaSH) is produced from hydrogen sulfide and sodium hydroxide shown in Equation (92):

| H_2_S + NaOH → NaSH + H_2_O | (92) |
| --- | --- |

Sodium nitrite (NaNO_2_) is produced in three steps from ammonia as shown in Equations (93) to (95):

| 4 NH_3_ + _5_ O_2_ → 4 NO + 6 H_2_O | (93) |
| --- | --- |
| 2 NO + O_2_ → 2 NO_2_ | (94) |
| NO_2_+ NO + 2 NaOH → 2 NaNO_2_ + H_2_O | (95) |

Thionyl chloride (SOCl_2_) is produced in two steps from sulphur shown in Equations (96) and (97):

| S + Cl_2_ → SCl_2_ | (96) |
| --- | --- |
| SCl_2_ + SO_2_ + Cl_2_ → 2 SOCl_2_ | (97) |

Trifluoroacetic acid (C_2_HF_3_O_2_) is produced in two steps from acetic acid shown in Equations (98) and (99)::

| C_2_H_4_O_2_ + 3 Cl_2_ → C_2_HCl_3_O_2_ + 3 HCl | (98) |
| --- | --- |
| C_2_HCl_3_O_2_ + 3 HF → C_2_HF_3_O_2_ + 3 HCl | (99) |

Start date: 01/01/2000

End date: 31/12/2019

Is data valid for entire period: True

Time period: Time of publications

Macro-economic scenario name: Business-as-Usual

Version: 3.0.8.0

Created: 7/28/2010 6:05:02 PM

Last edited: 9/22/2011 5:27:51 PM

Source: f0cbefe0-1979-4cb3-87c6-2bda57196b48_4c2b1cc3-84e5-4e35-b74e-8815eadbc674.spold

UUID: f0cbefe0-1979-4cb3-87c6-2bda57196b48
